# Effect of opioid-free anesthesia on the incidence of postoperative nausea and vomiting: A meta-analysis of randomized controlled studies

**DOI:** 10.1097/MD.0000000000035126

**Published:** 2023-09-22

**Authors:** Yanan Zhang, Dandan Ma, Bao Lang, Chuanbo Zang, Zenggang Sun, Shengjie Ren, Huayong Chen

**Affiliations:** a School of Anesthesiology, Weifang Medical University, Weifang, China; b Department of Anesthesiology, Yidu Central Hospital Affiliated to Weifang Medical University, Weifang, China; c Department of Anesthesiology, Weifang People’s Hospital, Weifang, China.

**Keywords:** extubation time, meta-analysis, opioid-based anesthesia, opioid-free anesthesia, postoperative analgesia, postoperative nausea and vomiting

## Abstract

**Background:**

Research on opioid-free anesthesia has increased in recent years; however, it has never been determined whether it is more beneficial than opioid anesthesia. This meta-analysis was primarily used to assess the effect of opioid-free anesthesia compared with opioid anesthesia on the incidence of postoperative nausea and vomiting.

**Methods:**

We searched the electronic databases of PubMed, the Cochrane Library, Web of Science and Embase from 2014 to 2022 to identify relevant articles and extract relevant data. The incidence of postoperative nausea and vomiting, time to extubation, pain score at 24 hours postoperatively, and time to first postoperative rescue analgesia were compared between patients receiving opioid-free anesthesia and those receiving standard opioid anesthesia. Differences in the incidence of postoperative nausea and vomiting were evaluated using risk ratios (95% confidence interval [CI]). The significance of the differences was assessed using mean differences and 95% CI. The heterogeneity of the subject trials was evaluated using the *I*^2^ test. Statistical analysis was performed using the RevMan 5.4 software.

**Results:**

Fourteen randomized controlled trials, including 1354 participants, were evaluated in the meta-analysis. As seen in the forest plot, the OFA group had a lower risk of postoperative nausea and vomiting than the control group (risk ratios = 0.41, 95% CI: 0.33–0.51, *P* < .00001; n = 1354), and the meta-analysis also found that the OFA group had lower postoperative analgesia scores at 24 hours (*P* < .000001), but time to extubation (*P* = .14) and first postoperative resuscitation analgesia time (*P* < .54) were not significantly different.

**Conclusions:**

Opioid-free anesthesia reduces the incidence of postoperative nausea and vomiting while providing adequate analgesia without interfering with postoperative awakening.

## 1. Introduction

Perioperative analgesia often relies on opioids, but it’s important to note that these medications can cause side effects, particularly at higher doses.^[1–3]^ Therefore, while the analgesic effects of opioids are being utilized, there is growing concern about their side effects. The most intense post-operative discomfort is pain, nausea, and vomiting, which can lead to aspiration, delayed wound healing, hematoma, bleeding, electrolyte imbalance due to dehydration, delayed recovery, and the ability to start eating or taking medication orally,^[4]^ all of which add to the cost of medical services. It is well known that perioperative administration of opioids tends to lead to continued opioid use, contributing to the current global opioid epidemic.^[5]^

Therefore, the use of opioids during surgery should be avoided, and perioperative opioids should be replaced by a combination of various non-opioids, nerve blocks or local anesthesia, and other modalities of multimodal analgesia to reduce the various adverse reactions that occur postoperatively due to perioperative opioid use. However, this recommendation has not been confirmed. Definitions of opioid-free anesthesia (OFAs) vary in the literature and between centers. Still, alpha 2 agonists such as dexmedetomidine and clonidine, ketamine, and lidocaine have been suggested as alternatives to opioids alone or in combination.^[6]^ The combination of intravenous lidocaine,^[7]^ ketamine,^[8]^ dexmedetomidine,^[9]^ and/or magnesium sulfate,^[10]^ a multimodal analgesic approach with important opioid retention effects, has been shown to reduce opioid-related adverse effects such as respiratory depression, urinary retention, nausea, vomiting, and intestinal obstruction.^[11]^ OFA is achieved precisely by the combination of these drugs. In the article by Frauenknecht et al^[12]^ studying the effects of OFA on postoperative pain, it was mentioned that OFA was associated with a decreased incidence of postoperative nausea and vomiting (PONV).

We conducted a systematic review and meta-analysis to investigate the effect of OFA on the incidence of PONV. Our analysis was based on a clinically meaningful perspective to assess the confidence level of the benefit-risk ratio of OFA in the available data.

## 2. Methods

### 2.1. Data sources and search strategy

Two investigators searched PubMed, the Cochrane Library, Web of Science, and Embase databases for relevant studies published from 2014 to 2022 using the terms “opioid-free anesthesia,” “opioid-free,” and “anesthetics,” with no restrictions on language or state of publication.

### 2.2. Eligibility criteria

Randomized controlled trial comparing “opioid-free anesthesia” (OFA group) with “opioid-based anesthesia” (opioid-based anesthesia group). Trials that examined adults (defined as ≥ 18 years) undergoing surgery under general anesthesia. Included trials must assess “opioid-free anesthesia” regardless of the drug, dose, or combination of medications used. The only scenario was the complete absence of opioids during general anesthesia. Patients in both groups must be under standardized general anesthesia, and we included randomized controlled trials (RCTs) that assessed postoperative outcomes. Only studies that assessed the incidence of PONV were considered for inclusion. Trials were excluded if participants were taking opioids concomitantly for another condition.

### 2.3. Data extraction and quality assessment

Data were collected by 3 authors (Y.Z., D.M., and B.L.) from the original text or inferred from the graphs and entered into an Excel spreadsheet. The following data were collected: author information, year of publication, patient information, type of procedure, type of drug used, and regional analgesic technique. If the full text of the article was not available, the authors were asked to provide the original text. Three authors independently assessed the risk of bias for each included study in 7 dimensions according to the Cochrane Risk of Bias Assessment Tool for RCTs.

### 2.4. Definition of primary and secondary outcome parameters

The common primary outcome was the incidence of nausea and vomiting within 24 hours postoperatively. Most studies did not distinguish between nausea and vomiting and reported both results. Since few patients present with vomiting without nausea, the incidence of postoperative nausea and PONV are usually quite similar^[13]^; therefore, when both outcomes of nausea and vomiting were recorded separately in the article, we used the incidence of postoperative nausea as the incidence of PONV.

Secondary outcomes were time to extubation (time to extubation defined as the time between cessation of drug infusion and endotracheal extubation), time to first postoperative rescue analgesia, and pain scores at 24 hours postoperatively (pain scores reported as visual, verbal, or numeric rating scales were converted to a standardized 0–10 analog scale for quantitative assessment). The studies used to examine 24 hours postoperative pain scores were assessed using either the visual analog scale or the numeric rating scale. On both scales, 0 indicated no pain and 10 indicated the most severe pain that was unbearable.

### 2.5. Data synthesis and analysis

For dichotomous data, we extracted the number of people at the event and the total number of participants; for continuous data, we collected means and standard deviations. If only the median and range were reported, the mean and standard deviation using the method described by Wan et al.^[14]^ We calculated the 95% confidence interval (CI) for the risk ratio (RR) for dichotomous data and 95% CI for the mean difference (MD) for continuous data. We expected heterogeneity between the data (as different populations and types of surgery were included); therefore, we used a random effects model. Heterogeneity was assessed using *I*^2^ statistics (*I*^2^ > 50% indicates substantial heterogeneity). Sensitivity analyses were also performed using a single deletion of a study to assess the stability of the results. Finally, summary analysis was visualized with forest plots, and statistical significance was set at *P* < .05.

## 3. Results

**Figure 1.**
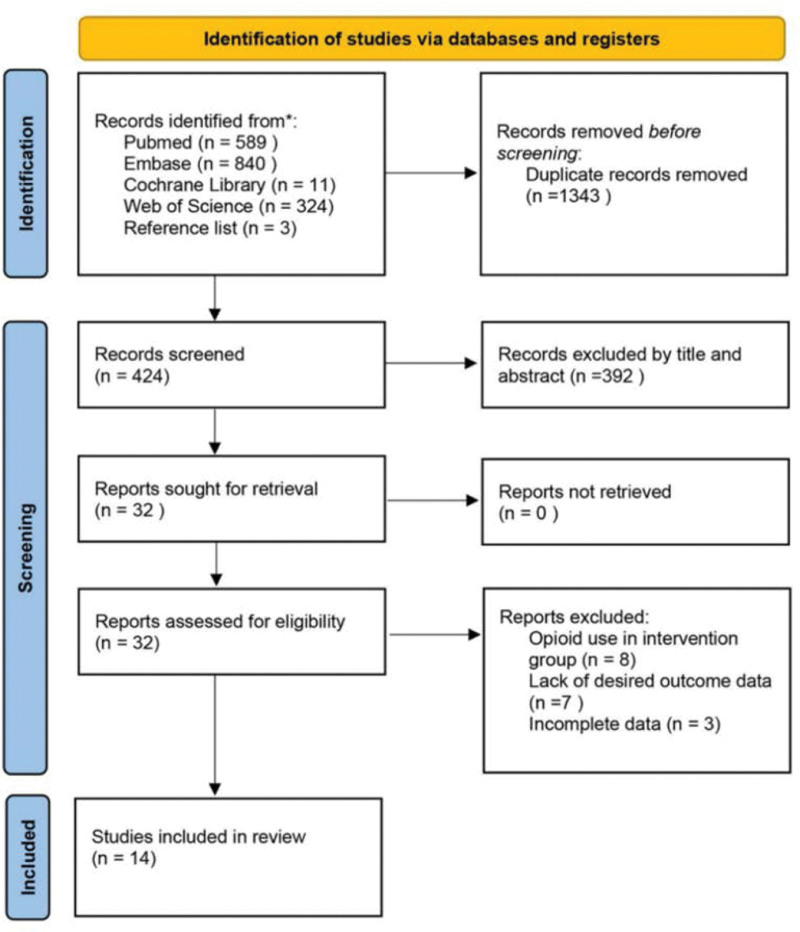
Flow diagram of included and excluded trials.

Figure 1 illustrates the study selection process. A total of 1766 articles were screened through the database and by reviewing the reference lists of relevant articles, 1343 were excluded due to duplication, 392 articles were excluded after review of titles and abstracts, and 18 of the remaining 32 articles were excluded for various reasons after careful reading of the full text, resulting in 14 RCTs being included in the current study. These studies included 14 articles published between 2014 and 2022 and recruited a total of 1354 participants (Fig. 1). Table 1 summarizes the characteristics of the 14 studies. Participants ages ranged from 30 to 75 years, and the BMI in the studies ranged from 23 to 60 kg/m^2^. Four RCTs specifically recruited female patients undergoing breast cancer or gynecologic surgery,^[15–18]^ and the other 10 studies included surgical procedures for both male and female patients.^[19–28]^

**Table 1:** Characteristics of studies (n = 14).

Studies	Age (yr)	Gender (M/F)	BMI (kg/m^2^)	Surgery	Opioid-free group	Control group	Anesthetic maintenance	Primary outcome
Soudi AM 2022	39.77 ± 11.3 vs 35.1 ± 9.4	5/26 vs 6/24	49.54 ± 6.87 vs 50.38 ± 8.25	Laparoscopic bariatric surgery	a, c	Fentanyl	Isoflurane	①②③
Aguerreche C 2021	69 ± 8 vs 71 ± 6	25/15 vs 31/9	26.99 ± 4.77 vs 26.23 ± 3.38	Cardiac surgery	a, b, c, e	Remifentanil, morphine	Propofol	①②
Bakan M 2014	43.1 ± 10.6 vs 43.8 ± 9.3	12/28 vs 13/27	27.2 ± 3.9 vs 28.9 ± 4.1	Laparoscopic cholecystectomy	a, b	Fentanyl, remifentanil	Propofol	①②
Bhardwaj S 2019	46 ± 8 vs 46 ± 12	22/18 vs14/26	35 ± 4 vs 37 ± 5	Laparoscopic urological procedures	a, b, c	Fentanyl	Propofol	①②④
Choi EK 2017	48.7 ± 12.4 vs 46.9 ± 10.5	9/31 vs 8/32	24 vs 25	Thyroidectomy	a	Remifentanil	Sevoflurane	①②③
Di Benedetto P 2021	59.36 ± 10.16 vs 56.40 ± 7.60	0/38vs 0/51	26.23 ± 4.79 vs 25.04 ± 6.09	Breast cancer surgery	c, e, f	Remifentanil, morphine	Desflurane or propofol	①③
Elshafie MA 2022	58.20 ± 6.38 vs 58.20 ± 6.38	10/10 vs 7/13	30.36 ± 3.99 vs 29.56 ± 11.17	Hepatic resection	a, b, e, g	Fentanyl	Sevoflurane	①
An G 2022	53.1 ± 8.6 vs 52.5 ± 10.2	28/23 vs 22/28	24.4 ± 3.1 vs 24.6 ± 2.9	Laparoscopic radical colectomy	a, d, g	Sufentanil, remifentanil	Sevoflurane	①②③
Hakim KK 2019	35.8 ± 6.7 vs 36.5 ± 7.2	0/40 vs 0/40	28.9 ± 4.1 vs 27.2 ± 3.9	Gynecological laparoscopic surgery	a	Fentanyl	Propofol	①②④
Ibrahim M 2022	29 ± 11 vs 32 ± 12	19/32 vs 21/31	45 ± 2 vs 44 ± 2	Laparoscopic bariatric surgery	a, b, c , g	Fentanyl	Sevoflurane	①②④
Mulier H 2020	53.92 ± 2.76 vs 50.32 ± 1.75	0/55 vs 0/149	25.01 ± 0.83 vs 25.33 ± 0.65	Breast cancer surgery	a, b, c	Remifentanil, sufentanil	Propofol*	①
Ziemann-Gimmel P 2014	50.3 ± 13.7 vs 50.4 ± 12.4	21/39 vs 16/43	44.15 ± 7.46 vs 45.32 ± 6.97	Bariatric surgery	a, c	Fentanyl	Sevoflurane or desflurane	①
Tripathy S 2018	52.7 ± 20.5 vs 55.4 ± 18.8	0/24 vs 0/24	23.3 ± 4.8 vs 22.5 ± 3.8	Breast cancer surgery	g	Morphine	Isoflurane	①③
Urvoy B 2021	NA	NA	NA	Hip replacement	a	Sufentanil	Sevoflurane	①②

Propofol*: Anesthesia maintenance in the opioid-free group was combined with intravenous and inhalation anesthesia.

① = incidence of postoperative nausea and vomiting, ② = extubation time, ③ = pain score at 24 h postoperatively, ④ = time to first postoperative need for rescue analgesia, a = dexmedetomidine, b= lidocaine, c = ketamine, d = ketorolac, e = MgSO4, f = clonidine, g = regional nerve block.

**Figure 2.**
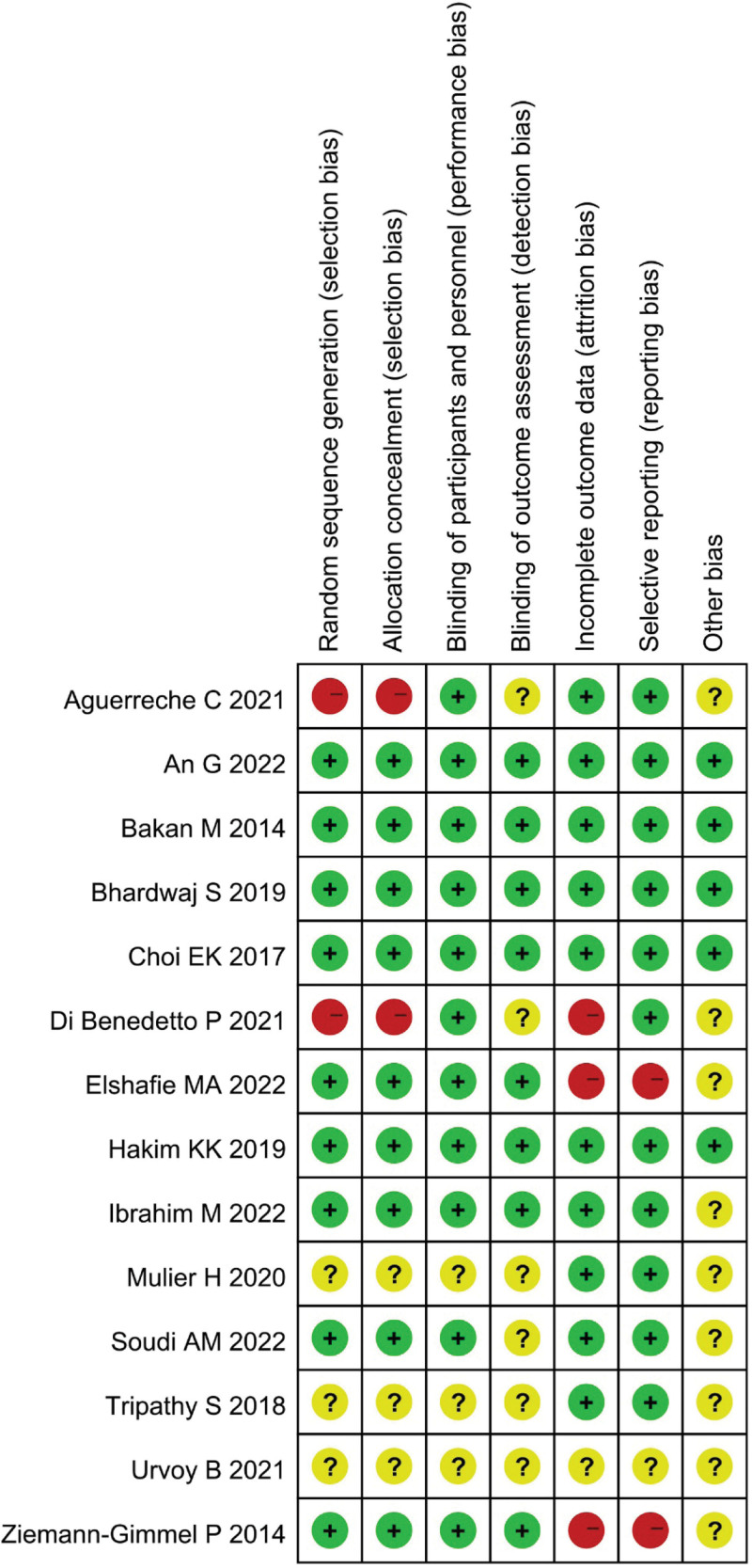
Risk of bias summary of included trials: evaluation of bias risk items for each included study. Green circle, low risk of bias; red circle, high risk of bias; and yellow circle, unclear risk of bias.

In the control group, 6 studies used fentanyl alone, and the remaining 8 mostly combinedremifentanil, sufentanil, or morphine. In the opioid-free group, 6 non-opioid analgesics, including dexmedetomidine, ketamine, magnesium, lidocaine, ketorolac, and clonidine, as well as local nerve blocks, were used in 1 trial with anesthesia using inhaled isoflurane under nerve block to maintain spontaneous breathing, while the other 13 studies used two or more drugs or combined local nerve blocks instead of opioids. The results of the risk of bias assessment are shown in Figure 2. The risk of bias was low in most of the trials. Five trials described the randomization process in detail and had a low overall risk of bias. Three studies^[16,17,25]^ lacked specific processes and data related to the trial, and the overall bias was unclear with some concerns. Attempts to communicate with all the 3 authors did not provide detailed data.

### 3.1. Primary outcome

#### 3.1.1. Incidence of nausea and vomiting within 24 hours after surgery.

**Figure 3.**
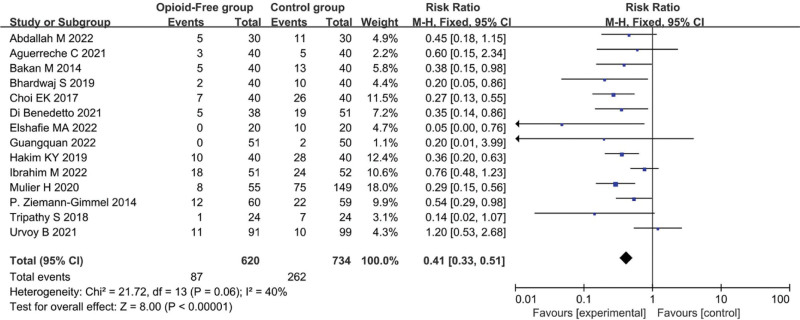
Forest plot comparing the risk of PONV. PONV = postoperative nausea and/or vomiting.

The OFA group had a lower risk of PONV than the control group (RR = 0.41, 95% CI: 0.33–0.51, *P* < .00001; n = 1354) with medium heterogeneity (*I*^2^ = 40%) (Fig. 3), and sensitivity analysis showed consistent findings (Table S1, Supplemental Digital Content, http://links.lww.com/MD/J912).

### 3.2. Secondary outcomes

#### 3.2.1. Extubation time.

**Figure 4.**
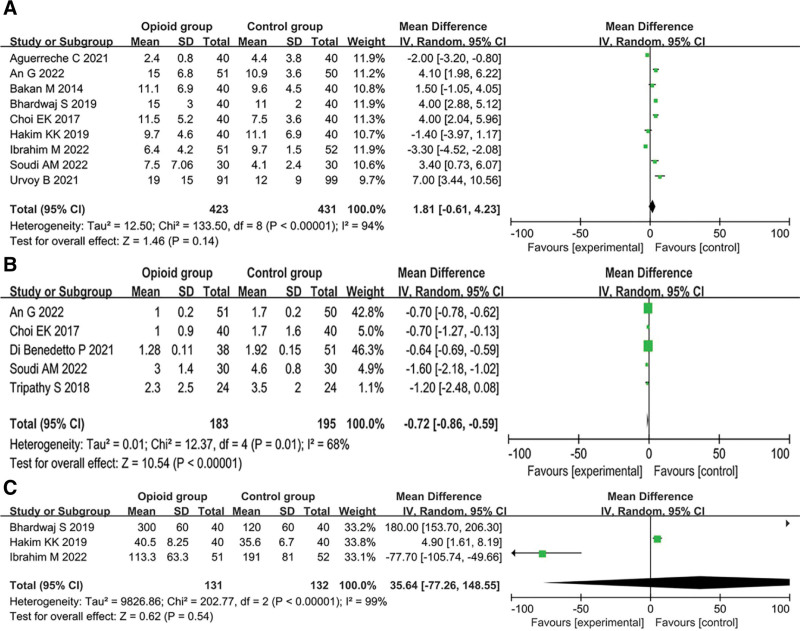
Forest plot comparing the risk of (A) extubation time, (B) pain score at postoperative 24 h, and (C) time of first rescue analgesia after operation.

Our findings showed no significant difference in extubation time between the OFA and control groups (MD = 1.81 minutes, 95% CI: −0.61 to 4.23, *P* = .14, *I*^2^ = 94%, 263 participants) (Fig. 4A). Sensitivity analysis showed that when 1 study was removed,^[28]^ the time to extubation was greater in the OFA group than in the control group (Table S2, Supplemental Digital Content, http://links.lww.com/MD/J913).

#### 3.2.2. Pain score at 24 hours postoperatively.

The pooled results showed that the OFA group had significantly lower pain scores than the control group (MD = −0.72, 95% CI: −0.86 to −0.59, *P* < .00001; 5 RCTs; n = 378) with moderate heterogeneity (*I*^2^ = 68%) (Fig. 4B), and the sensitivity analysis showed consistent findings (Table S3, Supplemental Digital Content, http://links.lww.com/MD/J915).

#### 3.2.3. Time to first postoperative need for rescue analgesia.

The pooled results showed no difference in the time to first rescue analgesia between the OFA and control groups. (MD = 35.64 minutes, 95% CI: −77.26 to 148.55, *P* = .54; 3 RCTs; n = 263), with high heterogeneity (*I*^2^ = 99%) (Fig. 4C), and the sensitivity analysis showed consistent results (Table S4, Supplemental Digital Content, http://links.lww.com/MD/J918).

### 3.3. Subgroup analysis

Due to the heterogeneity in our primary outcome (*I*^2^ = 41%), we performed a subgroup analysis of the primary outcome according to the type of surgery (laparoscopic or other open surgery), type of opioid used (fentanyl or other opioid), and type of anesthesia maintenance (intravenous maintenance or inhalation maintenance) to assess potential sources of heterogeneity.

**Figure 5.**
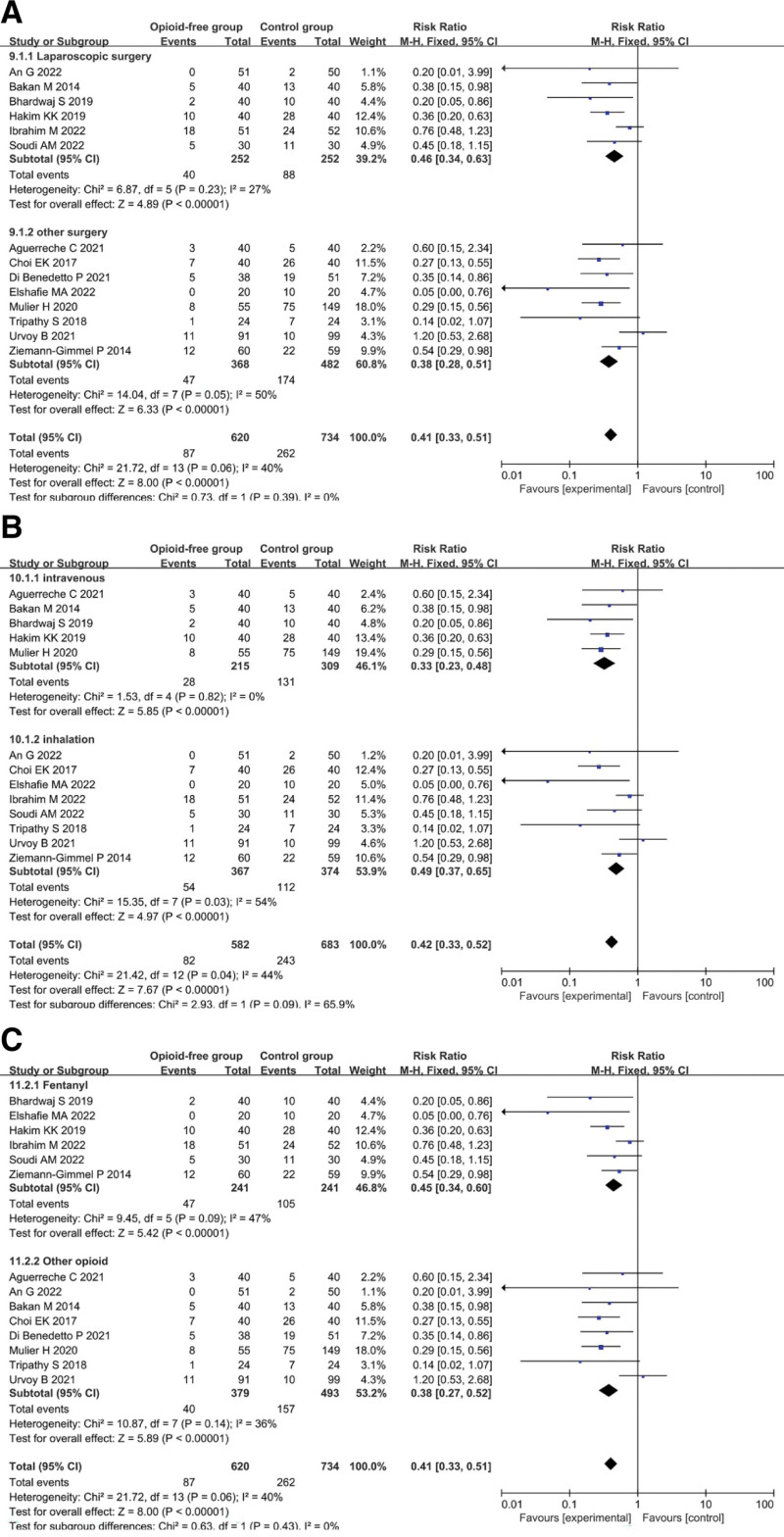
Subgroup analysis according to (A) the type of surgery, (B) the type of opioid used, and (C) the type of anesthesia maintenance.

Subgroup analysis according to the type of surgery showed that PONV was significantly lower in the opioid-free group than in the opioid group for both laparoscopic surgery and other types of surgery (Fig. 5A).

Subgroup analysis according to the type of anesthesia maintenance (intravenous or inhalation) showed that PONV was significantly lower in the opioid-free group than that in the opioid group, regardless of the type of anesthesia maintenance used (Fig. 5B).

A subgroup analysis based on the type of opioid used in the perioperative period showed that the incidence of PONV was significantly lower in the opioid-free group than in the opioid group, regardless of whether fentanyl or other opioids were used (Fig. 5C).

### 3.4. Publication bias

**Figure 6.**
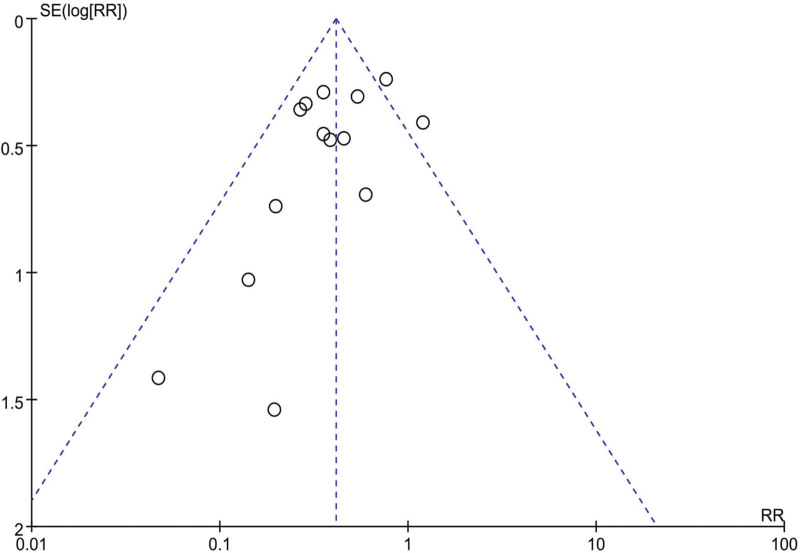
Funnel plot evaluating publication bias.

Figure 6 shows the publication bias funnel plot based on the primary outcome (PONV). The funnel plot for all included studies showed an asymmetric distribution around the effect estimate, indicating a slight publication bias in this analysis.

## 4. Discussion

In recent years, there has been a growing but consistently contradictory body of research on OFA. Most studies have concluded that OFA can provide the same analgesic effect as opioid anesthesia and reduce the adverse events associated with opioid anesthesia. However, a study by Beloeil et al^[29]^ showed that dexmedetomidine balanced with OFA did not reduce postoperative opioid-related adverse events. In contrast, it leads to a higher incidence of serious adverse events. In contrast, Schnabel et al^[30]^ concluded that patients treated with perioperative dexmedetomidine had reduced RR for nausea, vomiting, or PONV in the post anesthesia care unit or after 4 hours postoperatively. Similar to a previous meta-analysis by Hung et al,^[31]^ an analysis of the 4 studies that they retrieved showed a lower risk of PONV in the OFA group than in the control group (RR = 0.44). This is consistent with our results; however, the sample size of Hung et al was small, for which we included 14 clinical trials to study the effect of OFA on PONV.

The primary purpose of this meta-analysis was to compare the effects of opioid-free and opioid anesthesia on the incidence of PONV. Our meta-analysis of 14 studies found that intraoperative use of OFA was significantly associated with a reduced incidence of postoperative PONV, as well as reduced pain scores at 24 hours postoperatively, but had no significant effect on time to extubation or time to first postoperative rescue analgesia, confirming that OFA is a favorable option for reducing these adverse events and has no adverse effect on postoperative pain intensity or patient safety, which would provide a more favorable option for certain patient populations. For example, women are generally at a higher risk for PONV, suggesting that OFA may be particularly beneficial in breast surgery and gynecologic procedures.^[32]^ A meta-analysis by Salomé et al^[33]^ concluded that OFA does not provide clinically significant improvements in pain control. A study by Kelly^[34]^ identified a minimum clinically significant difference of 0.9 points in visual analog scale pain scores, below which, although statistically significant, is unlikely to have a clinical impact. In our meta-analysis, although pain scores at 24 hours postoperatively were lower in the opioid-free group than in the opioid group (i.e., a MD of 0.72 points), the difference was less than 0.9 points, indicating that it was not clinically significant. This also strengthens the credibility of our findings. In the present meta-analysis, OFA in most of these studies used dexmedetomidine, lidocaine, and ketamine to replace opioids in certain surgical procedures. Dexmedetomidine is an ɑ2-adrenergic agonist that inhibits the release of norepinephrine, leading to reduced excitation of the central nervous system, especially in the locus coeruleus,^[35]^ and Choi et al^[36]^ concluded that ɑ2-adrenergic agonists have significant analgesic effects and, although less analgesic than opioids, have been reported to reduce opioid requirements by 30% to 50%.^[37]^ The use of ketamine was previously restricted due to its associated side effects; however, in recent years, this drug has regained importance due to the discovery of superior analgesic abilities in new areas of research.^[38–42]^ A meta-analysis by Wang et al^[43](p)^ showed that perioperative intravenous ketamine reduced opioid doses and postoperative pain scores. In addition, Brinck et al^[8]^ concluded that ketamine use is associated with a reduction in the incidence of PONV. The OFA group in the randomized trials we included was made possible by these very non-opioids. These findings provide better theoretical support for the results of our meta-analysis. This provides a good option for perioperative multimodal analgesia. However, with the use of high doses of these drugs, the adverse events associated with these drugs may also increase. The literature on complete intraoperative opioid avoidance remains scarce. However, evidence now exists to support opioid-sparing strategies, as recommended in national guidelines. What is unclear is the added value of complete intraoperative opioid avoidance (e.g., reduction in postoperative opioid-related adverse events). Therefore, more studies are needed to investigate whether OFA is more effective and causes fewer adverse events than opioid-based anesthesia. Mulier and Dillemans^[44]^ concluded that when a multimodal analgesic approach is used, intraoperative opioids are not used at all while maintaining the patient’s perioperative hemodynamic stability and allowing rapid awakening after anesthesia. This finding is consistent with the results of our meta-analysis. Although PONV is often considered an unavoidable effect of opioid analgesia, a study by Macario et al^[45]^ showed that among postoperative pain and all other measures of discomfort, vomiting ranked highest among the outcomes that patients wanted to avoid. Therefore, we believe that OFA protocols are a major advantage in strategies to prevent PONV and should be considered, especially in high-risk patients.^[46]^

A limitation of this meta-analysis is that the number of studies may need to be larger to draw firm conclusions, and more studies are needed to support our conclusions. In addition, the evidence is weakened by the high degree of heterogeneity resulting from the differences in OFA protocols and surgical approaches (i.e., laparoscopic surgery or open surgery). Differences in perioperative opioid dosing owing to total anesthesia time and duration of surgery for different types of procedures can also have an impact on our observed metrics. Second, the drugs used to prevent and treat PONV (various antiemetics) also have an impact on our results. Third, regarding the association of OFA with the quality of postoperative recovery, our results cannot provide useful information on this issue because only 2 RCTs^[15,28]^ were available for analysis.

## 5. Conclusions

In conclusion, our analysis showed that OFA reduced the incidence of PONV compared to opioid-based anesthesia and provided the same effect as opioid anesthesia in terms of anesthetic awakening and postoperative analgesia. Not only the anesthetic component but also the characteristics of the surgical procedure and dietary recovery may lead to PONV.^[47]^ Therefore, postoperative prevention, treatment, and care for nausea and vomiting should include more effective and long-term programs. We believe that these results will help anesthesiologists develop individualized anesthetic strategies according to specific situations.

## Author contributions

**Conceptualization:** Yanan Zhang, Dandan Ma, Huayong Chen.

**Data curation:** Yanan Zhang, Dandan Ma, Shengjie Ren.

**Formal analysis:** Yanan Zhang, Bao Lang, Chuanbo Zang, Zenggang Sun.

**Resources:** Shengjie Ren, Huayong Chen.

**Software:** Chuanbo Zang, Zenggang Sun.

**Writing – original draft:** Yanan Zhang.

**Writing – review & editing:** Dandan Ma, Bao Lang, Chuanbo Zang, Zenggang Sun, Shengjie Ren, Huayong Chen.

## Supplementary Material








