# Supplementary material for: Effect of opioid-free anesthesia on the incidence of postoperative nausea and vomiting: A meta-analysis of randomized controlled studies
Source: Medicine (Baltimore). 2023 Sep 22;102(38):e35126. doi: 10.1097/MD.0000000000035126 (PMC10519493; doi:10.1097/MD.0000000000035126)
Supplement: Supplementary file 1 [file medi-102-e35126-s001.doc]

Supplementary Table 1〡Sensitivity anlaysis on PONV

| Removing individual studies | RR | P | I^2^ |
| --- | --- | --- | --- |
| Soudi AM 2022 | 0.41(0.33,0.51) | P＜0.00001 | 45% |
| Aguerreche C 2021 | 0.41(0.33,0.51) | P＜0.00001 | 44% |
| Bakan M 2014 | 0.41(0.33,0.52) | P＜0.00001 | 45% |
| Bhardwaj S 2019 | 0.42(0.34,0.53) | P＜0.00001 | 41% |
| Choi EK 2017 | 0.43(0.34,0.54) | P＜0.00001 | 38% |
| Di Benedetto P 2021 | 0.42(0.33,0.52) | P＜0.00001 | 44% |
| Elshafie MA 2022 | 0.43(0.35,0.54) | P＜0.00001 | 36% |
| An G 2022 | 0.42(0.33,0.52) | P＜0.00001 | 44% |
| Hakim KK 2019 | 0.42(0.33,0.53) | P＜0.00001 | 43% |
| Ibrahim M 2022 | 0.37(0.29,0.47) | P＜0.00001 | 22% |
| Mulier H 2020 | 0.44(0.35,0.55) | P＜0.00001 | 38% |
| Ziemann-Gimmel P 2014 | 0.40(0.32,0.50) | P＜0.00001 | 44% |
| Tripathy S 2018 | 0.42(0.34,0.52) | P＜0.00001 | 41% |
| Urvoy B 2021 | 0.38(0.30,0.47) | P＜0.00001 | 25% |
