# Supplementary material for: Effect of opioid-free anesthesia on the incidence of postoperative nausea and vomiting: A meta-analysis of randomized controlled studies
Source: Medicine (Baltimore). 2023 Sep 22;102(38):e35126. doi: 10.1097/MD.0000000000035126 (PMC10519493; doi:10.1097/MD.0000000000035126)
Supplement: Supplementary file 2 [file medi-102-e35126-s002.doc]

Supplementary Table 2〡Sensitivity anlaysis on time to extubation

| Removing individual studies | MD | P | I^2^ |
| --- | --- | --- | --- |
| Soudi AM 2022 | 1.62(-0.99,4.24) | 0.22 | 95 |
| Aguerreche C 2021 | 2.33(-0.32,4.97) | 0.08 | 93 |
| Bakan M 2014 | 1.86(-0.80,4.51) | 0.17 | 95 |
| Bhardwaj S 2019 | 1.50(-0.10,4.01) | 0.24 | 92 |
| Choi EK 2017 | 1.53(-1.08,4.15) | 0.25 | 94 |
| An G 2022 | 1.52(-1.08,4.13) | 0.25 | 94 |
| Hakim KK 2019 | 2.20(-0.42,4.83) | 0.10 | 95 |
| Ibrahim M 2022 | 2.47(-0.21,4.75) | 0.03 | 91 |
| Urvoy B 2021 | 1.25(-1.23,3.74) | 0.32 | 94 |
