# Supplementary material for: Effect of opioid-free anesthesia on the incidence of postoperative nausea and vomiting: A meta-analysis of randomized controlled studies
Source: Medicine (Baltimore). 2023 Sep 22;102(38):e35126. doi: 10.1097/MD.0000000000035126 (PMC10519493; doi:10.1097/MD.0000000000035126)
Supplement: Supplementary file 3 [file medi-102-e35126-s003.doc]

Supplementary Table 3〡Sensitivity anlaysis on 24h postoperative pain scores

| Removing individual studies | MD | P | I^2^ |
| --- | --- | --- | --- |
| Soudi AM 2022 | -0.66(-0.70,-0.62) | P＜0.00001 | 0% |
| Choi EK 2017 | -0.66(-0.87,-0.58) | P＜0.00001 | 76% |
| Di Benedetto P 2021 | -0.66(-1.43,-0.51) | P＜0.00001 | 69% |
| An G 2022 | -0.66(-1.45,-0.47) | P＜0.00001 | 73% |
| Tripathy S 2018 | -0.72(-0.86,-0.58) | P＜0.00001 | 74% |
