# Supplementary material for: Effect of opioid-free anesthesia on the incidence of postoperative nausea and vomiting: A meta-analysis of randomized controlled studies
Source: Medicine (Baltimore). 2023 Sep 22;102(38):e35126. doi: 10.1097/MD.0000000000035126 (PMC10519493; doi:10.1097/MD.0000000000035126)
Supplement: Supplementary file 4 [file medi-102-e35126-s004.doc]

Supplementary Table 4〡Sensitivity anlaysis on the timing of the first postoperative need for rescue analgesia

| Removing individual studies | MD | P | I^2^ |
| --- | --- | --- | --- |
| Bhardwaj S 2019 | -35.18(-116.09,45.73) | 0.39 | 97 |
| Hakim KK 2019 | 51.20(-201.34,303.74) | 0.69 | 99 |
| Ibrahim M 2022 | 91.94(-79.65,263.54) | 0.29 | 99 |
